# Optimizing Antiemetic Support in Anthracycline-Based Chemotherapy for Early Breast Cancer: Protocol for a Prospective Observational Study of Four-Drug Antiemetic Therapy Including Fosnetupitant and Olanzapine

**DOI:** 10.2196/85648

**Published:** 2026-02-03

**Authors:** Ayako Higuchi, Yumiko Koi, Tomoaki Eto, Yuka Maeda, Wakako Tajiri, Junji Kawasaki, Sayuri Akiyoshi, Hideki Ijichi, Yoshiaki Nakamura, Chinami Koga, Mototsugu Shimokawa, Hiroaki Shimizu, Toshihiro Matsumoto, Eriko Tokunaga

**Affiliations:** 1 Department of Pharmacy, National Hospital Organization Kyushu Cancer Center, Fukuoka, Japan; 2 Department of Breast Oncology, National Hospital Organization Kyushu Cancer Center, 3-1-1 Notame Minami-ku, Fukuoka, 811-1395, Japan, 81 092-541-3231; 3 Department of Pharmacy, National Hospital Organization Kokura Medical Center, Kitakyushu, Japan; 4 Department of Biostatistics, Yamaguchi University, Yamaguchi, Japan; 5 Department of Pharmacy, National Sanatorium Kikuchi Keifuen, Koshi, Japan

**Keywords:** antiemetics, olanzapine, Neurokinin-1 Receptor Antagonists, patient reported outcome measures, breast neoplasms

## Abstract

**Background:**

Prophylaxis for chemotherapy-induced nausea and vomiting (CINV) in patients receiving highly emetogenic chemotherapy (HEC) is essential. Four-drug antiemetic therapy, consisting of a neurokinin-1 receptor antagonist (NK1RA), a 5-hydroxytryptamine type 3 receptor antagonist (5-HT3RA), dexamethasone (DEX), and olanzapine (OLZ), is currently recommended for HEC. However, the efficacy, optimal dosing schedule, and appropriate dosage of OLZ remain unclear when combined with a highly selective NK1RA, fosnetupitant (FosNTP).

**Objective:**

This study aimed to evaluate the efficacy and safety of a four-drug antiemetic regimen including FosNTP and OLZ for prophylaxis of CINV in patients receiving anthracycline-based (neo)adjuvant chemotherapy for early breast cancer (EBC) and to explore outcomes with OLZ dose adjustments in the second cycle.

**Methods:**

This single-institution, prospective observational study will enroll 100 patients with EBC who undergo HEC. All patients will receive a four-drug antiemetic regimen consisting of FosNTP, 5-HT3RA, DEX, and the guideline-recommended dose of OLZ administered orally from days –1 to 4. During the second cycle of treatment, OLZ dosing may be adjusted based on tolerability and patient preference. The primary endpoint is the proportion of patients who experienced no nausea during the overall phase (0‐120 h) of the first cycle, as assessed using the daily visual analog scale. A sample size of 86 was calculated to assess the efficacy of the four-drug antiemetic regimen, including OLZ, assuming an expected no nausea rate of 42%, a threshold no nausea rate of 27% based on historical data, 90% power, and a 1-sided significance level of 5.0%. A single-sample z-test with a normal approximation will be used for the analyses.

**Results:**

This study was approved by the Ethics Committee of the National Hospital Organization Kyushu Cancer Center, and participant recruitment and data collection commenced in May 2024. As of September 2025, approximately 70 participants have been recruited. Data collection is expected to continue until April 2026, and both data collection and analyses are anticipated to be completed in 2027.

**Conclusions:**

This study will provide real-world evidence on the effectiveness and safety of a four-drug antiemetic regimen, including FosNTP and OLZ, in patients receiving anthracycline-based HEC and may inform optimal OLZ dosing strategies.

## Introduction

Chemotherapy-induced nausea and vomiting (CINV) is one of the most common and distressing side effects of cancer treatment, significantly impairing patients’ quality of life (QOL), especially due to nausea [1-3]. For highly emetogenic chemotherapy (HEC), international and Japanese guidelines currently recommend a four-drug antiemetic therapy consisting of a neurokinin-1 receptor antagonist (NK1RA), 5-hydroxytryptamine type 3 receptor antagonist (5-HT3RA), dexamethasone (DEX), and olanzapine (OLZ) [4-7]. OLZ is a multireceptor antipsychotic that exerts antiemetic effects partly via 5-HT2B/2C receptor antagonism and enhancement of ghrelin signaling [8]. However, owing to its broad receptor activity, OLZ frequently causes daily somnolence, particularly at the standard dose of 10 mg, with reported rates as high as 73.0%. Across guidelines and clinical studies, OLZ dosing for HEC prophylaxis generally ranges from 5 to 10 mg/day, reflecting a balance between efficacy and tolerability [5]. Based on previous studies demonstrating comparable efficacy and favorable tolerability of 5 mg OLZ relative to the standard 10 mg dose [9-12], the recommended initial dose in Japan is 5 mg/day [7]. Nevertheless, even at a dose of 5 mg, 20.3% of patients required dose reduction and 6.2% discontinued OLZ because of adverse effects [3,13]. In addition, a phase III randomized trial demonstrated that a 2.5 mg dose of OLZ was noninferior to 10 mg for CINV prevention, with significantly less somnolence [14].

Fosnetupitant (FosNTP) is a relatively new NK1RA that has demonstrated noninferiority to fosaprepitant in two phase III trials conducted in Japan (CONSOLE [15] and CONSOLE-BC [16]). In routine clinical practice, fosaprepitant has practical limitations, including admixture incompatibility with PALO and the risk of injection site reactions. FosNTP addresses several of these limitations and is therefore increasingly used in clinical practice [16]. However, evidence regarding the combined use of FosNTP with OLZ for CINV prophylaxis remains limited [17].

This study will evaluate the real-world efficacy and safety of a four-drug regimen (FosNTP, palonosetron [PALO], DEX, and OLZ) for CINV prophylaxis in patients receiving (neo)adjuvant anthracycline-based chemotherapy (HEC) for early breast cancer. The primary objective is to estimate the proportion of patients who experience no nausea during the overall phase (0‐120 h) in the first chemotherapy cycle. A secondary objective is to explore clinical outcomes among patients who require OLZ dose adjustments after the first cycle.

## Methods

### Study Setting

This prospective, single-institution, observational cohort study will be conducted at the National Hospital Organization Kyushu Cancer Center, Japan. At our institution, FosNTP-based antiemetic prophylaxis is used in routine care for anthracycline-based HEC, and the use of alternative NK1RAs (eg, fosaprepitant) is limited due to practical concerns such as admixture incompatibility with PALO and injection site reactions. As a result, establishing a concurrent comparison group receiving another NK1RA is not feasible; therefore, we selected a prospective observational cohort design to evaluate the real-world efficacy and safety of the FosNTP-containing four-drug regimen and to explore OLZ dose adjustments. This study was designed as a prospective observational cohort study, and all antiemetic treatments, including OLZ dose adjustments, were provided as part of routine clinical practice at the discretion of the treating physicians; the study protocol did not assign or mandate any intervention. We aim to enroll 100 participants over 2 years from May 2024 to April 2026. The first participant was enrolled on May 7, 2024.

### Study Participants

#### Inclusion criteria

Patients eligible for this study must meet all the following criteria:

1. Female patients 20‐74 years old who were able to understand and provide their written informed consent
2. A diagnosis of early-stage or locally advanced breast cancer (stages I–III) in a patient scheduled to receive (neo)adjuvant chemotherapy
3. No prior chemotherapy
4. A plan was made to receive either EC (epirubicin 90 mg/m^2^ and cyclophosphamide 600 mg/m^2^ every 3 weeks) or dose-dense EC (epirubicin 90 mg/m^2^ and cyclophosphamide 600 mg/m^2^ ever weeks)
5. An Eastern Cooperative Oncology Group performance status (ECOG PS) of 0 or 1
6. A preserved major organ function within 8 days prior to enrollment (defined as AST<100 IU/L, ALT<100 IU/L, and total bilirubin <2.0 mg/dL [34.2 µmol/L])
7. Able to understand and report patient-reported outcomes (PROs)
8. Clinical data available for analyses

#### Exclusion Criteria

Patients excluded for this study must meet any the following criteria:

1. A history of allergy to any of the study drugs or related compounds
2. Vomiting or nausea within one day prior to enrollment
3. Use of antiemetic drugs within two days prior to enrollment
4. A history of, or scheduled to undergo surgery or radiation therapy within seven days prior to enrollment or during the observation period
5. Regular use of medications that may affect antiemetic efficacy, including selective serotonin reuptake inhibitors (SSRIs), serotonin-norepinephrine reuptake inhibitors (SNRIs), serotonin-dopamine antagonists, multireceptor-targeted antipsychotics, corticosteroids, histamine H_1_ receptor antagonists, phenothiazine antipsychotics, dopamine receptor antagonists, and benzodiazepines
6. Initiation of opioid analgesics within 14 days prior to enrollment
7. Seizure disorders requiring anticonvulsant treatment
8. Gastrointestinal obstruction, including pyloric stenosis or intestinal obstruction
9. Pregnant or lactating women or women of childbearing potential unwilling to use effective contraception
10. A history of diabetes requiring treatment with insulin or oral hypoglycemic agents
11. Concurrent participation in another interventional clinical trial
12. Any condition that, in the opinion of the attending physician, would render the patient unsuitable for participation in the study

### Procedure and Treatment

The study consisted of a screening period prior to the initiation of EC, followed by a treatment period of 7 days (168 h), beginning at the start of EC administration. Written informed consent will be obtained prior to enrollment after confirming that the patients meet all the inclusion criteria and do not meet any exclusion criteria.

All patient information will be handled in a reidentifiable anonymized format, with the linkage key strictly controlled and never shared externally. Data will be securely managed within the institution and securely stored for at least five years after study completion or three years after publication, whichever is longer.

### Treatment

All enrolled patients will receive PALO (day 1: 0.75 mg intravenously, 30 min before chemotherapy), FosNTP (day 1: 235 mg intravenously, 30 min before chemotherapy), DEX (day 1: 9.9 mg intravenously, 30 min before chemotherapy), and OLZ 5 mg. The initial dose of FosNTP, PALO, and DEX follows the approved dosing in Japan and the recommended antiemetic regimen for HEC in current guidelines [4-7]. OLZ was initiated at 5 mg/day as the recommended initial dose in Japan, considering the balance between antiemetic efficacy and tolerability [7,9-13]. In this study, OLZ will be administered orally and post-prandially from day –1 to day 4, rather than initiating administration on the evening of day 1 as is conventional [9]. This earlier initiation was adopted to ensure adequate plasma concentrations at the start of treatment based on its oral pharmacokinetics, with a time to maximum plasma concentration of approximately 6 hours and an elimination half-time of about 30 hours [18]. Given the higher incidence of acute-phase CINV on days 1‐2 reported for HEC [19] , initiating OLZ on the evening of day –1 was intended to provide sufficient and sustained drug exposure at the onset of treatment and throughout the early high-risk period for optimal antiemetic control.

For the second cycle, the OLZ dose may be adjusted based on patient preference after the physician reviews tolerability with attention to clinically relevant adverse events such as somnolence in the first cycle. Based on this assessment, patients may choose to continue with 5 mg/day, reduce the dose to 2.5 mg/day, increase it to 10 mg/day, or discontinue OLZ. The reason for the second cycle OLZ dose decision is documented. The selected OLZ dose will be confirmed directly with the patient before the start of the second cycle. Any OLZ dose reduction or discontinuation during a cycle will be determined by the physician in consultation with the patient, considering patient preference and clinical tolerability.

If nausea or vomiting occurs and the physician deems it necessary, or if the patient requests additional antiemetic therapy, rescue medications not included in the study protocol may be administered. These include metoclopramide, domperidone, alprazolam, and other drugs, as appropriate.

### Follow-Up

During the seven-day treatment period, all patients will record symptoms in a diary, including nausea, vomiting, use of rescue medications (additional antiemetics), daytime somnolence, and its impact on daily activities. Nausea, somnolence, and the impact of somnolence on daily activities will be assessed using a visual analog scale (VAS). The impact of somnolence on daily activities is defined as interference with usual daily activities.

**Figure 1.**
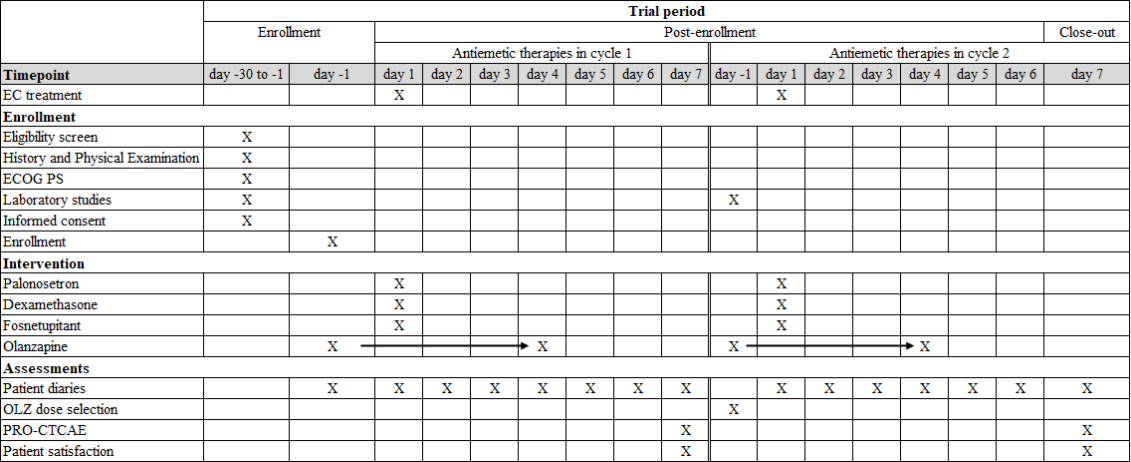
The schedule of enrollment, intervention, and assessments. EC: epirubicin plus cyclophosphamide; ECOG PS: Eastern Cooperative Oncology Group performance status; OLZ: Olanzapine; PRO-CTCAE: Patient-Reported Outcomes version of the Common Terminology Criteria for Adverse Events. X indicates the timing of planned procedures, interventions, or assessments. Repeated Xs or arrows across consecutive days indicate repeated daily administration/assessment during the indicated period.

At the end of the observation period, patients will retrospectively assess the adverse events they experienced over a seven-day period using the Patient-Reported Outcomes version of the Common Terminology Criteria for Adverse Events (PRO-CTCAE) [20]. They will also assess their satisfaction with antiemetic therapy using a seven-point Likert scale: very satisfied, satisfied, somewhat satisfied, slightly satisfied, slightly dissatisfied, dissatisfied, or very dissatisfied. Figure 1 illustrates the timeline for participant enrolment, intervention, and assessment.

### Endpoints

- Primary endpoint: The primary endpoint is no nausea during the overall phase (0‐120 h after the start of epirubicin administration) in the first cycle. This binary endpoint was selected to reflect the clinically meaningful goal of nausea control, consistent with endpoint definitions commonly used in clinical trials evaluating antiemetic efficacy in the CINV/HEC setting [5,21]. Nausea is assessed using a 100-mm VAS, and no nausea is operationally defined as a VAS score of <5 mm, as described in the Definitions of key endpoints section. Continuous VAS scores will also be summarized as supportive information.

- Secondary endpoints: The secondary endpoints include no nausea during phases other than the overall phase of the first cycle, complete response (CR) rate, complete control (CC) rate, total control (TC) rate, time to treatment failure, degree of daytime somnolence, impact of somnolence on daily activities, no nausea during the second cycle, rate of OLZ dose modification, incidence of adverse events, and patient satisfaction with antiemetic therapy.

### Observation Periods

The observation periods will be classified into five phases: acute (0‐24 h after the start of epirubicin administration), delayed (24‐120 h), overall (0‐120 h), extended delayed (24‐168 h), and extended overall (0‐168 h).

### Definitions of Key Endpoints

In this study, no nausea is defined as a VAS score of <5 mm. VAS is widely used to assess nausea and allows a more granular evaluation of nausea severity and changes over time compared with categorical rating scales [22]. Although no universally accepted VAS cutoff for nausea has been established, this threshold was selected based on previously published CINV studies involving anthracycline-treated breast cancer patients [23-25]. The CR rate is defined as not an emetic event, and no rescue medication will be administered. The CC rate showed no emetic event, no rescue medication, and no significant nausea (VAS score <25 mm). The TC rate is defined as no emetic events, rescue medication, or nausea. Time-to-treatment failure was defined as the time from the start of epirubicin administration to the earliest occurrence of either the first episode of vomiting or the first use of rescue medication.

### Patient and Public Involvement

The patients and public were not involved in the development of the research question, study design, outcome measures, or recruitment plans.

### Statistical Considerations

#### Sample Size Justification

Previous studies have reported varying rates of no nausea during the overall phase depending on the antiemetic therapy used. A 3-drug combination of FosNTP, PALO, and DEX reportedly achieved a no nausea rate of 27.5% in patients receiving HEC [26], and this value was used as the historical control threshold in the current study. In addition, a phase 3 randomized controlled trial by Navari et al showed that adding 10 mg of OLZ to a 3-drug combination of aprepitant, PALO (5-HT3RA), and DEX improved the rate of nausea by 15.4% compared to the antiemetic regimen without OLZ [27]. Based on this evidence, a threshold no-nausea rate of 27% was defined for this study, especially in the context of using a lower and potentially more tolerable dose of OLZ (5 mg). The required sample size was calculated using an expected a no-nausea rate of 42%, a minimum acceptance rate of 27%, a one-sided significance level of 5.0%, and a power of 90%. A one-sided significance level was selected because the primary objective is to demonstrate that the no nausea rate exceeds a prespecified historical threshold, and only an improvement over the threshold is clinically meaningful. Based on these assumptions, the required number of participants was 86. The target sample size was set at 100 patients, considering the anticipated consent and valid response rates.

### Statistical Analyses

The primary analysis will be conducted on the full analysis set, defined as patients who have undergone at least one efficacy assessment. Patients who were retrospectively deemed ineligible or registered in violation of the protocol will be excluded from this set in accordance with the predefined criteria. The primary endpoint is a proportion, and a two-sided 90% confidence interval will be estimated using a normal approximation. A z-test based on the normal approximation will be performed. If the result is statistically significant, the primary objective will be considered met. We will conduct prespecified exploratory stratified analyses by OLZ dose modification in the second cycle and established CINV risk factors. These analyses will be descriptive and hypothesis-generating. Missing diary entries and incomplete PRO-CTCAE responses will not be imputed. Analyses will be conducted using available data, and the extent of missingness will be reported. For the primary endpoint, participants without sufficient diary or VAS data to determine “no nausea” during the overall phase will be classified as not having achieved no nausea. All statistical analyses will be performed using R version 4.4.2 or later.

### Ethical Considerations

This study was approved by the Ethics Committee of the National Hospital Organization Kyushu Cancer Center (approval number: [2023‐41]). Written informed consent will be obtained from all the participants prior to enrollment, after the treating physician has explained the study and a study pharmacist has provided additional explanations. The study will be conducted in accordance with the Declaration of Helsinki and applicable national regulations. To protect participants’ privacy and confidentiality, all study data will be deidentified before analysis. Personal identifiers will be removed and replaced with unique study IDs. The linkage file will be stored separately on a secure, access-restricted system and will be accessible only to authorized study personnel. Where feasible, data will be reported in aggregate to minimize reidentification risk. No compensation will be provided to participants. The results will be disseminated through presentations at academic conferences and publications in peer-reviewed journals. No personally identifiable information is disclosed.

This study has been registered with the University Hospital Medical Information Network Clinical Trials Registry (UMIN-CTR) under the identifier UMIN000054250.

No ancillary or post-trial care is required, as the study involves routine chemotherapy and antiemetic treatment without additional risks beyond usual care.

## Results

This study was approved by the Ethics Committee in March 2024, and participant recruitment and data collection commenced in May 2024. As of September 2025, approximately 70 participants had been recruited. Data collection is expected to continue until April 2026, and both data collection and analyses are anticipated to be completed in 2027.

## Discussion

This study is expected to provide real-world evidence that a four-drug antiemetic regimen including FosNTP and OLZ offers clinically meaningful primary prophylaxis of CINV with acceptable tolerability in patients receiving perioperative epirubicin–cyclophosphamide chemotherapy for breast cancer. It also aims to inform the feasibility of OLZ dose reduction or discontinuation in subsequent cycles while maintaining CINV control. This protocol incorporates PROs as a direct measure of symptom burden. Despite adherence to current guideline-recommended regimens, approximately 70% of patients with breast cancer continue to experience insufficient control of nausea [5,28], underscoring a substantial unmet need in supportive care. Effective CINV prevention is essential for maintaining treatment adherence and enabling successful completion of (neo)adjuvant chemotherapy. Among patients receiving HEC, anthracycline-based chemotherapy is associated with an increased incidence of acute-phase CINV on days 1 and 2 when OLZ is not included in antiemetic therapy, highlighting the importance of earlier intervention [19]. In the present study, OLZ was administered from day –1 to day 4, a novel schedule that differs from the conventional initiation on the evening of day 1 and, to our knowledge, has not been previously evaluated in this population.

By assessing the real-world effectiveness of a four-drug antiemetic therapy incorporating FosNTP and OLZ, this study will generate clinically relevant data beyond those available from conventional approaches. Prior phase III trials conducted in Japan established the efficacy and safety of FosNTP-based prophylaxis in combination with PALO and DEX [15,16]; however, evidence on FosNTP in combination with OLZ remains limited. Furthermore, evaluating the tolerability of guideline-recommended OLZ dosing and exploring dose adjustments in subsequent cycles may inform individualized antiemetic strategies that balance efficacy and safety.

A key strength of this study is the prospective collection of PROs, enabling a detailed assessment of nausea control and tolerability in routine practice.

This study has several limitations. As a single-arm observational study, it is inherently limited in its ability to support causal inference, and residual confounding cannot be excluded. In particular, patient characteristics and clinical decision-making may have influenced both OLZ dose adjustments and outcomes. Accordingly, the results should be interpreted descriptively as real-world associations rather than as evidence of causal effects.

These findings are expected to refine antiemetic practice by providing real-world evidence on optimized four-drug therapy, improving supportive care standards, and enhancing the quality of life and treatment adherence for patients with early breast cancer receiving anthracycline-based chemotherapy. Future studies in broader settings will be valuable to confirm generalizability and further refine OLZ dosing strategies.

## Supplementary material

10.2196/85648 Checklist 1 SPIRIT 2025 checklist.

## Abbreviations

5-HT3RA: 5-hydroxytryptamine type 3 receptor antagonist
CC: complete control
CINV: chemotherapy-induced nausea and vomiting
CR: complete response
DEX: dexamethasone
EBC: early breast cancer
EC: epirubicin and cyclophosphamide
PS: Eastern Cooperative Oncology Group performance status
HEC: highly emetogenic chemotherapy
NK1RA: neurokinin-1 receptor antagonist
OLZ: olanzapine
PALO: palonosetron
PRO: patient-reported outcomes
PRO-CTCAE: Patient-Reported Outcomes version of the Common Terminology Criteria for Adverse Events
QOL: quality of life
TC: total control
VAS: visual analog scale
